# Cough and dyspnea management in pulmonary fibrosis

**DOI:** 10.1097/SPC.0000000000000753

**Published:** 2025-03-25

**Authors:** Allard van Veelen, Marlies S. Wijsenbeek, Thomas Koudstaal

**Affiliations:** Department of Pulmonary Medicine, Erasmus University Medical Center, Rotterdam, The Netherlands

**Keywords:** chronic cough, dyspnea, palliative care, pulmonary fibrosis, symptom management

## Abstract

**Purpose of the review:**

Pulmonary fibrosis (PF) is characterized by relentless scarring of the lungs, declining lung function, and increasing symptom burden. In PF, dyspnea and cough are the most common symptoms, severely impacting quality of life. This review highlights recent advances in understanding their mechanisms and explores evolving strategies for management of these symptoms.

**Recent findings:**

Advances in non-pharmacologic approaches, including hand-held fans, dyspnea services and pulmonary rehabilitation are playing a vital role in dyspnea management. Opioids, while effective in reducing exertional dyspnea in controlled settings, show limited benefit for daily life breathlessness and are associated with significant adverse events, highlighting the need for cautious, individualized use. For refractory cough, promising studies are investigating the role of opioids and neuromodulatory therapies. Non-pharmacologic approaches, including speech therapy, and behavioral interventions, provide complementary approaches. A multidisciplinary approach and individualized care plans to address the multifactorial nature of dyspnea and cough are key.

**Summary:**

Effective management of dyspnea and cough can importantly improve patients' quality of life. Further research is required to refine treatment protocols, optimize palliative care interventions, and identify and test novel therapeutics. Translation of these findings into clinical practice requires a focus on evidence-based, patient-centered care.

**KEY POINTS**

- Complete a comprehensive work-up in both dyspnea and cough to diagnose and possibly treat comorbidities, before classifying it as idiopathic pulmonary fibrosis.
- Non-pharmacological treatments, including handheld fans, pulmonary rehabilitation and cognitive behavioral therapy play a vital role in managing dyspnea and chronic cough.
- Early integration of palliative care with multidisciplinary collaboration is needed to improve quality of life and reduce hospitalizations.
- A personalized approach is needed to combine pharmacological and non-pharmacological with early palliative care, to optimize symptom control.

## INTRODUCTION

Pulmonary fibrosis (PF) is an irreversible disease characterized by excessive scarring of the lungs, resulting in impaired gas exchange, dyspnea, reduced quality of life, and frequently culminating in respiratory failure or death. Dyspnea and chronic cough are the most prevalent and burdensome symptoms experienced by patients [1,2]. These symptoms occur throughout the disease course but are particularly prominent in the advanced stages of the disease, where the primary focus of care transitions from modifying disease progression to alleviating symptoms and enhancing quality of life [3]. Evidence-based approaches to palliative care in PF are just emerging and urgently needed. The 2023 European Respiratory Society clinical practice guideline emphasizes the integration of palliative care for patients with PF, stressing the importance of addressing symptom burden, psychosocial support, and advance care planning [4]. A nationwide survey by Fujisawa *et al.* revealed significant variability in the implementation of palliative care practices for interstitial lung disease (ILD) patients among pulmonary specialists, underscoring the need for increased awareness and more standardized approaches [5]. While standardization through guidelines is essential to ensure equitable and high-quality care, it must be balanced with individualized interventions that address the specific needs of patients with PF. This includes consideration of disease trajectory, patient preferences, and broader cultural and systemic factors influencing care delivery [6,7].

This article provides a narrative review of the management of cough and dyspnea in PF, summarizing recent advancements in both pharmacologic and non-pharmacologic treatment strategies. While not a systematic review, the literature was selected through targeted searches of relevant studies, key clinical guidelines, recent trials, and expert recommendations. The review also underscores the increasing recognition of palliative care as a vital component of PF management, emphasizing the need for a multidisciplinary approach to alleviating symptom burden. It explores current guidelines, emerging evidence, and the challenges associated with implementing standardized yet individualized palliative care practices, as well as potential solutions to improve access and integration into routine care.

## CHRONIC COUGH: A PERSISTENT CHALLENGE IN PULMONARY FIBROSIS

**FIGURE 1.**
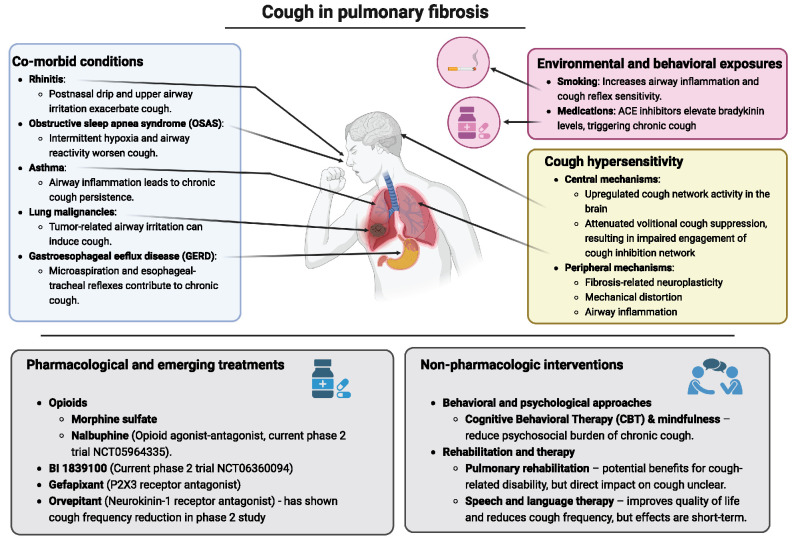
Contributing factors to chronic cough in pulmonary fibrosis and current (non-)pharmacological treatment options.

Chronic cough is a debilitating symptom in PF, affecting up to 85% of patients and profoundly impacting their quality of life [8,9]. The etiology of cough in PF is complex, multifactorial, and not yet fully understood. It involves the interplay of mechanical, neural, and inflammatory pathways, as well as environmental exposures and comorbid conditions [10,11]. These contributing factors, together with (non-)pharmacological treatment options are described in Fig. 1.

## UNDERLYING MECHANISMS IN COUGH

Environmental and behavioral exposures, including smoking and certain medications, contribute to cough in PF. Smoking is a well-known risk factor for pulmonary diseases and may exacerbate airway inflammation and cough reflex sensitivity [12,13]. Additionally, the use of angiotensin-converting enzyme (ACE) inhibitors, which are associated with increased bradykinin levels, can trigger chronic cough [14,15].

Next to that, several co-morbid conditions have been identified that may influence chronic cough in PF, including rhinitis, airway inflammation in asthma, obstructive sleep apnea syndrome (OSAS), and gastroesophageal reflux disease (GERD). Rhinitis, a common comorbidity, exacerbates cough through postnasal drip and upper airway irritation [16,17]. Airway inflammation, driven by cytokine and immune cell activity, contributes to cough chronicity [17,18]. OSAS, frequently comorbid in PF, may exacerbate cough through intermittent hypoxia and heightened airway reactivity [14]. Addressing these traits with targeted interventions, such as intranasal corticosteroids for rhinitis or continuous positive airway pressure (CPAP) for OSAS, could theoretically reduce cough in some patients and improve patient outcomes.

GERD is highly prevalent in PF and may play a role in exacerbating chronic cough through microaspiration and esophageal-tracheal reflexes [17]. The management of GERD in PF typically involves lifestyle modifications, proton pump inhibitors, and surgical interventions like fundoplication [19]. However, studies such as the PACIFY trial have not shown significant improvements in cough frequency or disease progression with GERD management alone [20▪]. Another study has shown an increase in non-acid reflux after acid suppression, which raises the question whether antacids should be used as much as they are in general practice [21].

Coughing is a common symptom in lung malignancies, which has a higher prevalence of 13.74% in patients with PF, which should always be taken into consideration in the work-up [22].

Another potential cause of persistent coughing is cough hypersensitivity. The current concept of cough hypersensitivity in PF is that it arises from both peripheral and central mechanisms. Mechanical distortion of lung tissue due to fibrosis activates rapidly adapting receptors (RARs) and other mechanosensitive pathways, leading to exaggerated coughing reflexes [23,24]. Additionally, the destruction of peripheral nerve fibers by fibrosis induces neuroplastic changes, resulting in central sensitization and heightened responsiveness to otherwise non-noxious stimuli [9,25,26]. Elevated expression of sensory receptors such as P2X3 has also been implicated in perpetuating cough hypersensitivity [27,28].

## DIAGNOSTIC WORKUP FOR COUGH IN PULMONARY FIBROSIS

A structured diagnostic approach is crucial for assessing and managing cough in PF. A detailed patient history and comprehensive physical examination aid in identifying treatable traits, such as airway inflammation, reflux-related symptoms, and environmental exposures, as well as comorbid conditions that may exacerbate cough. High-resolution computed tomography (HRCT), pulmonary function testing, and laboratory assessments play a critical role in identifying underlying contributors, including disease progression, infections, airway inflammation, GERD, and cough hypersensitivity.

## PHARMACOLOGICAL AND EMERGING TREATMENTS

Recent research has focused on novel pharmacological treatments that target specific mechanisms underlying chronic cough in PF. Opioids like morphine and nalbuphine show promise in reducing cough frequency in patients with idiopathic PF [29,30▪,^31^]. Nalbuphine, an opioid agonist-antagonist, has shown promise in reducing cough frequency, with the ongoing CORAL trial expected to provide further insights (NCT05964335) [30▪]. Another novel compound is currently being investigated to reduce cough in PF and modulate pulmonary inflammation and fibrosis (NCT06360094). Other agents, including sodium cromoglicate (PA101), azithromycin and RVT-1601, have shown negative results, underscoring the challenges in finding universally effective treatments [32,33].

Antifibrotics have shown some potential benefits in mitigating chronic coughing in idiopathic pulmonary fibrosis (IPF) [34▪,^35^]. Immunosuppression might also play a role in the treatment of chronic cough. High dosage of oral corticosteroids showed an improvement in coughing in patients with IPF in a small, non-randomised study [12]. However, one should be aware that using high dosage of immunosuppression in IPF is obsolete because of harmful effects [36]. In clinical practice a short trial of low dose steroids is common practice. In addition to that, patients treated with either mycophenolate or cyclophosphamide in SSc-ILD also reported an improvement in coughing [37].

Neuromodulatory medications can also help reduce coughing. Gabapentin, a neuromodulatory drug, has been shown to decrease both cough severity and frequency while improving quality of life in patients with chronic cough [38]. However, studies in PF are lacking. Another treatment option is gefapixant, a P2X3 receptor antagonist, which is approved for chronic cough. However, in a study involving patients with IPF, gefapixant did not significantly improve objective cough measures. Nevertheless, post-hoc analyses and secondary endpoints suggest a potential therapeutic benefit, warranting further investigation in patients with PF [27]. Orvepitant, a neurokinin-1 receptor antagonist, has also shown promise; in a phase 2 study, it was associated with a reduction in cough frequency and an improvement in quality of life [39].

## NON-PHARMACOLOGIC INTERVENTIONS

Non-pharmacologic strategies remain essential components of chronic cough management, though research specifically in PF is limited and many findings are extrapolated from chronic cough management. Cognitive behavioral therapy (CBT) and mindfulness-based interventions have shown potential in reducing the psychosocial burden of chronic cough by addressing emotional triggers and improving coping mechanisms [40,41]. Pulmonary rehabilitation programs, combining exercise, education, and psychosocial support, may offer additional benefits in managing cough-related disability, though their direct impact on cough requires further investigation [42]. Speech and language therapy may also have a positive effect on the quality of life and the number of coughs throughout the day. However, this effect is only seen up to a few weeks after therapy has ended [43].

Taken together, chronic cough in PF represents a multifaceted challenge requiring a holistic approach. Addressing exposures, treatable traits, and underlying pathophysiological mechanisms is critical for effective management. Emerging pharmacological therapies and non-pharmacologic interventions offer hope for improving outcomes, but further research is needed to validate these strategies.

## DYSPNEA: MECHANISMS AND MANAGEMENT

**FIGURE 2.**
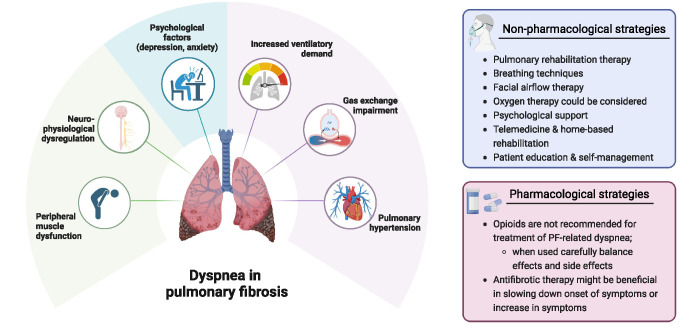
Contributing factors for dyspnea in pulmonary fibrosis and current (non-)pharmacological treatment options.

Dyspnea is the most prevalent symptom of PF, significantly impacting quality of life [44]. Management guidelines advocate a multidisciplinary approach integrating various strategies [4,42]. Dyspnea results from a complex interplay of physiological, neurological, and psychological factors. The following section reviews its pathophysiology, diagnostic approach, and treatment strategies, summarized in Fig. 2.

## PATHOPHYSIOLOGY: MECHANISMS DRIVING DYSPNEA

The primary mechanism driving dyspnea in PF is reduced lung compliance due to progressive fibrosis, which increases the mechanical workload of breathing [45]. This leads to greater respiratory effort and an increased ventilatory demand [46]. Additionally, fibrosis-induced alveolar-capillary disruption impairs gas exchange, resulting in hypoxemia, which further exacerbates dyspnea by stimulating chemoreceptors that drive heightened respiratory effort [47,48]. Pulmonary hypertension is a common comorbidity in advanced PF, contributing to right ventricular dysfunction, increased dead space ventilation, and worsening exertional dyspnea [49]. Peripheral muscle dysfunction, arising from chronic hypoxia and reduced physical activity, further exacerbates breathlessness by accelerating fatigue during exertion [50]. Neurophysiological mechanisms also play a crucial role. Fibrosis and inflammation can disrupt pulmonary afferent pathways, leading to heightened central perception of dyspnea [51]. This can make patients feel breathless even in the absence of significant desaturation or ventilatory limitation [52]. Psychological factors further amplify dyspnea perception, with anxiety and depression creating a vicious cycle in which breathlessness heightens psychological distress, which in turn intensifies the sensation of dyspnea. Understanding these interwoven mechanisms is essential for developing targeted interventions that address both the physiological and perceptual aspects of dyspnea in PF.

## DYSPNEA IN PULMONARY FIBROSIS; A COMPREHENSIVE DIAGNOSTIC APPROACH

A comprehensive dyspnea work-up in PF evaluates severity, contributing factors, and coexisting conditions [44]. Assessment begins with a detailed history of symptom onset, progression, and impact on daily life [53]. Identifying associated symptoms helps distinguish PF-related dyspnea from other causes [54]. Since dyspnea in PF may also result from coexisting conditions such as infections, cardiac conditions, thyroid disorders, obstructive sleep apnea syndrome (OSAS), pulmonary embolism, or pulmonary hypertension, these should be systematically evaluated during work-up. High-resolution computed tomography (HRCT) is the gold standard for diagnosis and disease monitoring, with attention for pulmonary vascular involvement and signs of pulmonary hypertension [49,55]. Pulmonary function tests, including FVC and DLCO, track disease severity, while blood gas analysis assesses hypoxemia and informs oxygen therapy decisions [46,56]. Echocardiography can be used to screen for pulmonary hypertension and right heart dysfunction, both of which are key contributors to dyspnea [49]. Exercise tests like the six-minute walk test (6MWT) evaluate functional capacity and oxygen desaturation, while cardiopulmonary exercise testing (CPET) can help to differentiate pulmonary from cardiovascular causes [50].

## MANAGING DYSPNEA IN PULMONARY FIBROSIS: A MULTIMODAL APPROACH

Management of dyspnea in PF requires a multimodal approach addressing both physiological and perceptual aspects of breathlessness. Pharmacologic strategies include antifibrotic therapy with nintedanib and pirfenidone, which slow disease progression and may help preserve lung function and mitigate increase of dyspnea [7]. Oxygen therapy improves exercise tolerance in patients with exertional or resting hypoxemia [57]. Opioids remain controversial due to limited evidence and potential risks, and current guidelines do not recommend their routine use [42]. Anxiolytics, including SSRIs and benzodiazepines, may help manage dyspnea-related anxiety [58]. However, a recent study did not show any benefit for mirtazepine in breathlessness, compared to placebo [59]. Therefore, the role of anxiolytics requires further study.

Non-pharmacologic interventions play a central role in dyspnea relief, particularly pulmonary rehabilitation, which improves endurance, reduces dyspnea, and enhances quality of life [50]. Modern rehabilitation incorporates exercise training, breathing techniques, education, and psychological support, tailored to individual patient needs [42]. Additionally, facial airflow therapy using a handheld fan stimulates trigeminal nerve receptors, helping to reduce breathlessness perception [60].

Addressing the psychological impact of dyspnea is equally important, as fear and anxiety can exacerbate breathlessness, limit activity, and reduce quality of life [61,62]. Panic-related dyspnea further reinforces avoidance behaviors, highlighting the need for psychological interventions such as CBT, which has shown promise in reducing anxiety and dyspnea-related distress in chronic lung diseases [63]. While data on CBT in PF are limited, its efficacy in COPD could also suggest potential benefits for PF patients.

As access to in-person rehabilitation remains challenging, telemedicine and home-based rehabilitation offer promising alternatives, particularly for patients with mobility limitations [64]. Early evidence suggests these remote programs improve adherence and provide comparable benefits to in-person rehabilitation [65]. Moving forward, integrating digital health solutions, psychological support, and personalized rehabilitation strategies will be key to optimizing dyspnea management in PF.

## MULTISTAKEHOLDER TRANSMURAL PALLIATIVE CARE IN PULMONARY FIBROSIS

Effective palliative care for PF requires multidisciplinary collaboration to enhance quality of life, reduce hospitalizations, and align end-of-life care with patient preferences. A structured, team-based approach involving pulmonologists, nurses, and respiratory therapists improves symptom management and increases home or hospice-based care [66,67].

Early integration of palliative care is both feasible and effective, addressing symptom burden, advance care planning, and caregiver support while reducing healthcare costs [67,68]. Expanding access through telemedicine and community-based initiatives is particularly beneficial for underserved populations [69]. Caregiver involvement remains crucial, with targeted interventions like counseling and shared decision-making reducing burden and enhancing support [70,71].

Transmural palliative care, bridging hospital and home settings, lowers hospital admissions and increases home-based end-of-life care, though challenges remain in aligning treatment goals across providers [72,73]. Decision aid tools further facilitate palliative care discussions and referrals, reinforcing the need for systematic integration into routine care [74]. While these models improve patient outcomes, further research is needed to refine implementation and address remaining barriers.

## BARRIERS TO IMPLEMENTATION

Patients with PF face substantial barriers to palliative care due to prognostic uncertainty, communication gaps, delayed referrals, and systemic limitations. The unpredictable disease course complicates timely palliative care initiation, while misconceptions and poor communication among patients, families, and providers further hinder access [69,75,76].

Pulmonologists report greater difficulty managing palliative care in IPF than in malignancies, partly due to a lack of standardized protocols and inconsistent symptom management strategies like opioid use for dyspnea [69]. Systemic barriers, including inadequate provider training and fragmented healthcare policies, further restrict access [68,77,78].

Overcoming these challenges requires earlier palliative care integration, better provider education, and improved communication. Policy reforms that expand access and strengthen interdisciplinary collaboration are essential. Future research should refine referral criteria, evaluate early interventions, and develop structured care pathways to ensure equitable palliative care delivery.

## FUTURE PERSPECTIVES AND DEVELOPMENT

The management of chronic cough and dyspnea in PF is evolving, but challenges remain in integrating emerging treatments into routine care. A personalized approach combining pharmacologic and non-pharmacologic strategies is key to optimizing symptom control. While novel therapies targeting neural hypersensitivity and inflammation show promise, their long-term efficacy and safety require validation. Precision medicine, guided by biomarkers, may help tailor treatments to individual patients. Non-pharmacologic interventions like pulmonary rehabilitation and supplemental oxygen remain underutilized. Expanding telehealth and remote rehabilitation programs could improve accessibility and proactive care. Multidisciplinary collaboration among pulmonologists, palliative care specialists, and allied health professionals is essential for a holistic approach that includes caregiver support and structured decision-making.

In summary, optimizing the management of chronic cough and dyspnea in PF requires an integrated approach that combines pharmacologic and non-pharmacologic strategies with early palliative care. Refining treatment pathways to target exposures, treatable traits, and underlying mechanisms will be key to improving symptom control. Emerging therapies and digital health tools offer promise but must be made more accessible. Strengthening multidisciplinary collaboration and standardizing care will be essential for advancing toward a more structured, equitable, and patient-centered model.
